# Urban Exotic Pollution: The Harmful Environmental Footprint for Health and Historical Architecture

**DOI:** 10.3390/ijerph20064715

**Published:** 2023-03-07

**Authors:** Cristina Postolachi, Alexandru Cocean, Silvia Garofalide, Bogdanel Silvestru Munteanu, Georgiana Cocean, Nicanor Cimpoesu, Vasile Pelin, Iuliana Cocean, Silviu Gurlui

**Affiliations:** 1 Atmosphere Optics, Spectroscopy and Laser Laboratory (LOASL), Faculty of Physics, Alexandru Ioan Cuza University of Iasi, 11 Carol I Bld., 700506 Iasi, Romania; 2 Laboratory of Applied Meteorology and Climatology, A Building, Physics, Research Center with Integrated Techniques for Atmospheric Aerosol Investigation in Romania, RECENT AIR, Alexandru Ioan Cuza University of Iasi, 11 Carol I, 700506 Iasi, Romania; 3 Rehabilitation Hospital Borsa, 1 Floare de Colt Street, 435200 Borsa, Romania; 4 Faculty of Material Science and Engineering, Gheorghe Asachi Technical University of Iasi, 59A Mangeron Bld., 700050 Iasi, Romania

**Keywords:** dust binders, aluminate, anti-skid, coagulants/flocculants, cleaning induced water contamination process

## Abstract

The study in this paper was carried out as a result of the observation of pollution phenomena and foaming effects associated with anthropogenic activities, including street cleaning activity. The processes of dust binding used in order to reduce PM10 and PM 2.5 pollution has been proven to be inefficient, and even contributing to pollution with particulate matter. Our results suggest that the use of dust binders must be integrated in a technique that includes methods of removing agglomerated particle structures resulting from the process of coagulation or flocculation. These are the conclusions of the investigations carried out by spectroscopic methods (FTIR, SEM-EDX) on samples collected from the streets of Iasi on 10 March 2021, and on samples collected from the surface of the Precinct Wall of the historical monument Golia—Iasi Monastery Ensemble (Romania). On the later samples, coloristic analysis was also performed. The alert for investigation was given by the foaming waters that were leaking on the streets. The phenomenon was observed after the streets had been washed by specialized vehicles. Analyses revealed compounds used as dust binders and coagulant type (aluminum sulfate, sodium aluminate and their derivatives, plus anti-skid chemicals such as calcium chlorine and magnesium chlorine), as well as organic compounds included in aggregate type structures, and they showed contamination of the Golia Precinct Wall. The results show that the dust binders or coagulants used as such, or embedded in various products intended for the cleaning process of streets or other outdoor public places, must be subject to regulation. Otherwise, there is a risk of adding more pollutants during an operation with the opposite purpose. The migration of these pollutants on the studied building offers an image on how both our health and all constructions and equipment exposed in the open air are affected.

## 1. Introduction

Over time, through air quality measurements and monitoring, chemical compounds specific to the urban area have been detected, including, from case to case, specific local influences. However, there are instances where exotic pollutants are detected in the atmosphere. These are chemical species that are not usually found in the atmosphere, leading to an unusual pollution episode such as the one with dust binder compounds that we will refer to in the current study. The source of pollution with dust binders is also an unusual one, being generated by the attempt to find a solution whose purpose is precisely to reduce dust pollution, but which, through faulty administration, can lead to effects that, on the contrary, increase pollution and add new compounds to the already existing ones. With road dust being identified as an important contributor to urban pollution, with micro particles such as PM 10 and PM 2.5, studies have been required to find solutions to this issue. One solution is to form aggregate structures that would no longer present the danger of easily dispersion into the atmosphere under the influence of natural air currents or induced by anthropogenic activities. This led to the use of chemical compounds that were already known for their coagulating or flocculating properties, and generically referred to as dust binders [1,2]. Thus, the proposed technique is not limited to the formation of larger particles under the action of solutions of coagulating chemical compounds, but also their subsequent removal by vacuum suction. In fact, the basis for reducing the pollution with PM 10 and PM 2.5 induced in the air by the presence of dust on the streets is the process of removing it (most efficiently by vacuum suction), and the formation of larger particles is necessary to streamline the process, thus avoiding releases of micro particles during the process of street cleaning and garbage handling. The removal of newly formed aggregate particles by the dust binding process is absolutely necessary, otherwise in time, due to the friable characteristics, it will be crushed under the mechanical action of various factors, with the process being influenced by temperature, humidity, light, etc. At the same time, monitoring is required accompanied by the analysis of the effects generated by the use of coagulants in order to avoid additional damage to the environment involving both human health and urban civil buildings [3].

Dust binding agents are part of the category of coagulants/flocculants already used in water treatment and pre-treatment stations. Of these, sodium aluminate in its various forms (NaAlO_2_, Na_2_O·Al_2_O_3_, Na_2_Al_2_O_4_, or as hydrated form NaAl(OH)_4_) and aluminum hydroxide (Al(OH)_3_) are most often used in water treatment, and also in construction for the coagulation of cements [4,5,6,7]. The polluting effects of infiltration of these compounds into the water used for domestic purposes have been reported [8]. Another category of coagulants used for wastewater treatment and taken as dust binders are calcium chloride and magnesium chloride. These had already been used as anti-skid during the winter, and even mixed with sand.

The spread of the particles resulted from material outcome in the dust binding process is studied herein, using aluminum as a tracking marker. The particles deposition is studied on a historical building, the Precinct Wall of Golia Monastery of Iasi (Romania).

## 2. Materials and Methods

Following the effects of water leaks with foam on the streets of Iasi as a result of their washing with specialized technical means, samples were collected (denoted as “Street Samples” herein) on 10 March 2021, which led to the identification of compounds classified as dust binders and anti-skid compounds. Because the phenomenon continued to be noticed, on 14 April 2021, a study of the influences of pollution on the Precinct Wall of the historical monument Golia Monastery Ensemble of Iasi (denoted as “Golia Wall” herein) began. Due to similarities in the spectral analysis, and the fact that the building named “Golia Wall” faces one of the streets where the event of foaming water on 10 March 2021 took place, the two studies were combined into one. The aim was to verify the migration of compounds from those foamed waters and their influence on the building in discussion. This also provides an image of how other buildings and/or equipment exposed in the street areas can be affected by particle deposition during or as a result of such dust binding operations carried out on city streets.

**Figure 1:**
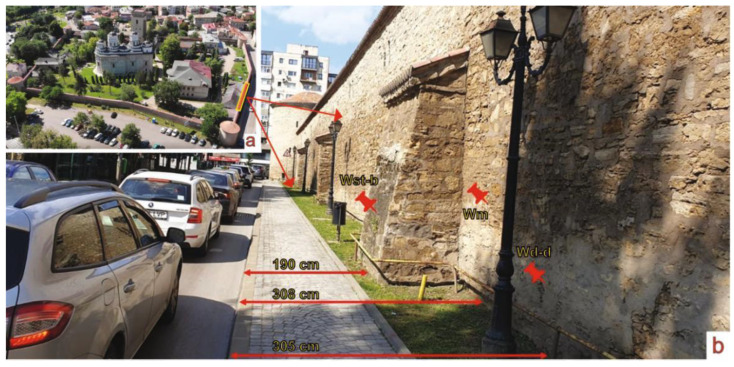
Image of the general view of the Precinct Wall of Golia Monastery in Iasi and West sampling area position (**a**) and the West side sampling area and the sampling spots denoted as Wst-b, Wd-d, Wm (**b**).

**Figure 2:**
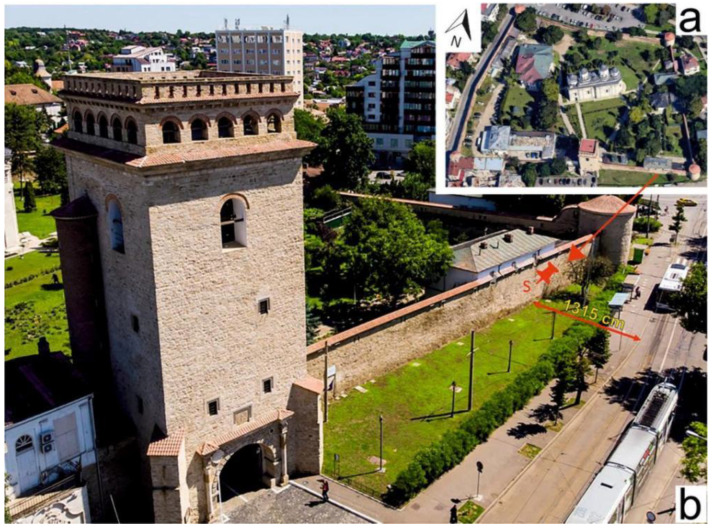
Image of the general view of the Precinct Wall of Golia Monastery in Iasi and South sampling area position (**a**) and the South side sampling area and the sampling spot denoted as S (**b**).

Samples were taken from the walls of the Golia monastery in the western and southern areas, as shown in the images in Figure 1a,b and Figure 2a,b. The distances from the wall to the street in the sampling areas are listed in Table 1, and show the direct exposure to traffic influence, especially on the western side.

The water samples from the streets of Iasi collected just after the washing operation had been performed showed abundant and persistent foam floating on the water. The foam was collected using a spoon, and deposited on a tray of a 16 cm^2^ area for drying at room temperature of 20–22 °C. From the remaining liquid, 5 mL was poured in a similar 16 cm^2^ area tray and dried at room temperature (about 22 °C). The drying of the materials resulting from the samples was carried out until the mass was constant between two weighings. Powder samples resulting from light scraping at several spots of the Precinct Wall of Golia Monastery (identified further as “Golia Wall samples” and “Golia Wall sampling spots”) were collected in separate containers using a brush. The solid material resulted from both foam and liquid after drying, and the powder of Golia Wall samples were analyzed by Fourier Transform Infrared Spectroscopy (FTIR) and Energy Dispersive X-Ray Spectroscopy (EDX) to identify chemical components. For FTIR analyses, the samples were mixed with potassium bromide (KBr) and then pressed at 100 atm pressure in a stainless steel ring to form a pellet of clear appearance. The pellet in the stainless steel ring was placed in the FTIR sample holder. Morphology of the dry materials resulted after water evaporation, and the powder of Golia Wall samples were studied by imaging means of Scanning Electron Microscopy (SEM). A Bomem MB154S FT-IR spectrometer at an instrumental resolution of 4 cm^−1^ (Bomem, ABB group, Canada) and Vega Tescan LMH II, Brno, Cehia, were used for the FT-IR and SEM-EDX investigation, respectively. Colorimetric determinations were performed on the Golia wall sampling spots using the device NR10QC Universal Chromatic Meter Color-Difference Meter Color Analyzer (Shenzhen 3nh Technology Co., Ltd., Shenzhen, China.

The meteorological context is also analyzed together with the size of the particles in the atmosphere for the period of time that is the subject of the study, using online resources of meteorological history [9] and AERONET data [10]. 

## 3. Results and Discussion

### 3.1. Chemical Composition and Morphology of the Collected Samples

**Figure 3:**
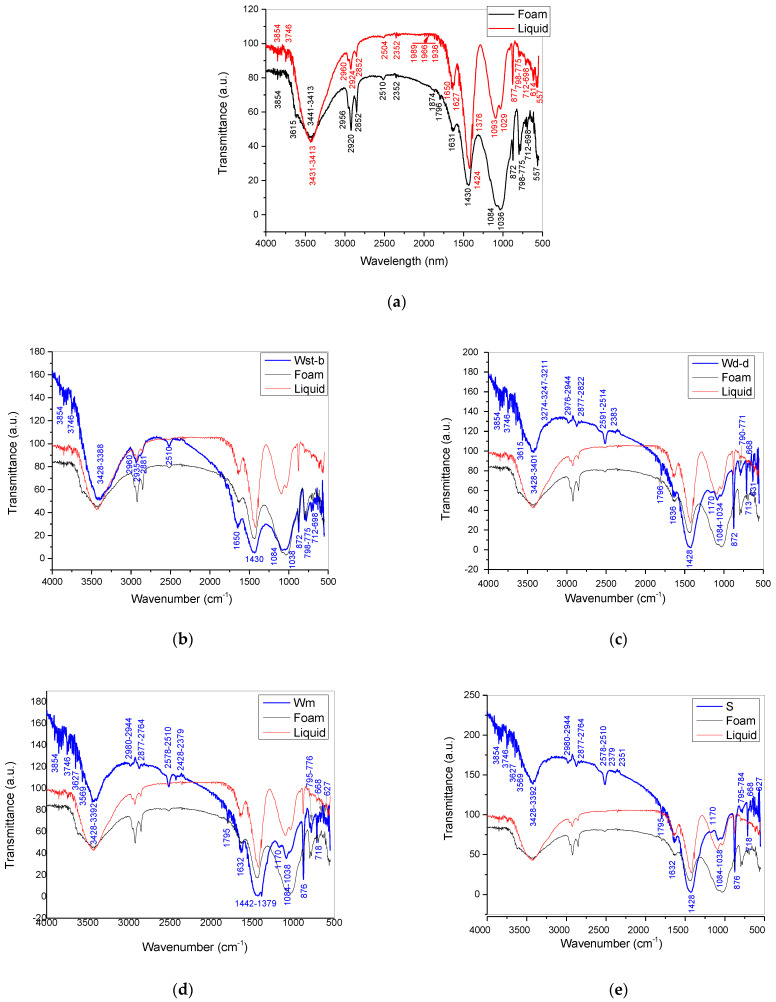
FTIR spectra of solid material resulting after drying the foam (black line) and after drying the liquid (red line) (**a**); and spectra of Golia Wall samples (Wst-b, Wd-d, Wm and S) compared with the street samples of foam and liquid (**b**–**e**).

FTIR spectra (Figure 3a) of the solid material resulting from foam and liquid after drying show similarities, but also differences that are further presented in Table 2 in the form of functional groups assigned to the vibrational modes identified in the spectra. Also presented in Table 2 are the functional groups identified based on the vibrational modes from the spectra of the Golia Wall samples in Figure 3b–e, which are presented compared to the foam and liquid samples. Based on Table 2 and Figure 3, the results, with the similarities and differences resulting from the spectral analysis, are further discussed.

**Figure 4:**
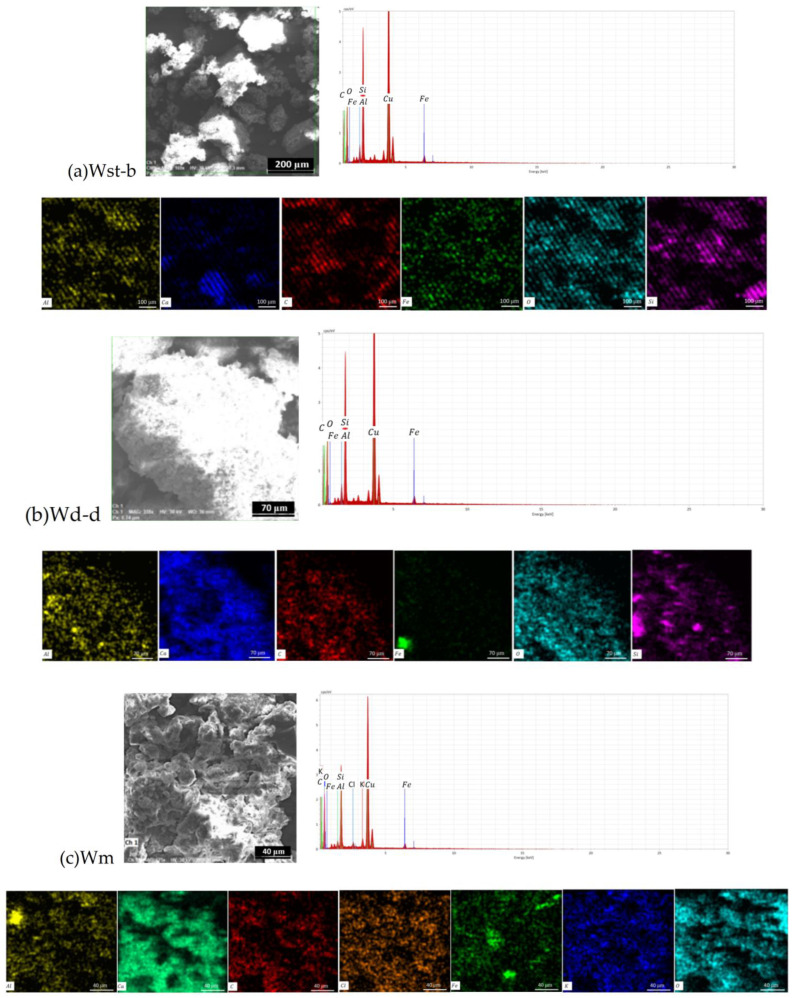

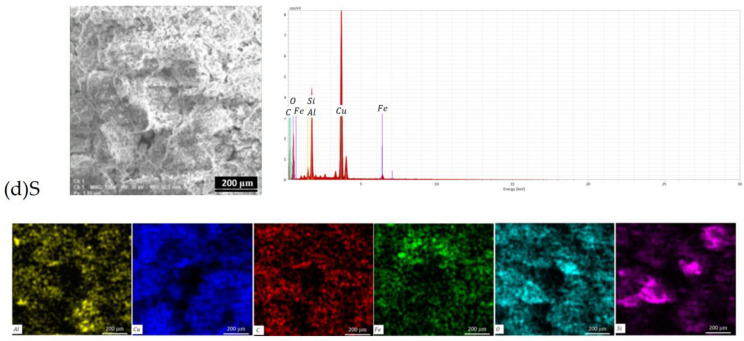
Elements mapping on the SEM images of Golia Wall samples: Wst-b (**a**); Wd-d (**b**); Wm (**c**); and S (**d**).

**Figure 5:**
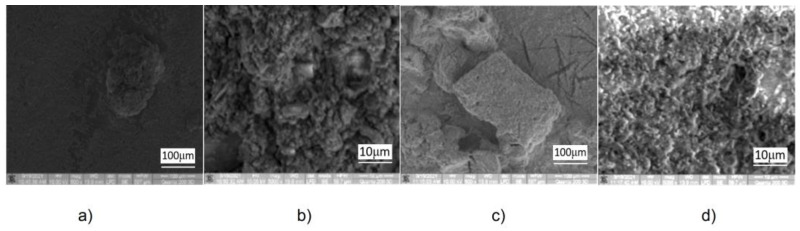
SEM images of 500 (**a**,**c**) and 5000 magnitude (**b**,**d**) of the solid material resulting after liquid drying (**a**,**b**) and foam drying (**c**,**d**).

The FTIR spectra of the solid components separated by evaporation from foam and washing liquid (Figure 3a) denote a mixture of chemical compounds, which of interest for the present investigation is the presence of Al-OH bonds and sulfate groups assigned to aluminates (NaAlO_2_, Na_2_O·Al_2_O_3_, or Na_2_Al_2_O_4_ or alkali form in solution, NaAl(OH)_4_) and aluminum sulfate (Al_2_SO_4_), respectively. Aluminate-type compounds, but also others based on aluminum, such as aluminum sulfate, are used based on the property of aluminum ions to coordinate with organic compounds, but also with some inorganic compounds [3,8]. Particles of dispersed or powdered substances are thus collected in agglomerations that could then be filtered from water or aspirated in a solid state from surfaces. Complementary to the FTIR analysis, the results of the EDX analysis (Figure 4) also provided information about the presence of calcium chloride (CaCl_2_), an ionic compound that cannot be detected by IR vibration modes. The two elements (Ca and Cl) are in atomic percentages, corresponding to the stoichiometry of the molecule. Calcium chloride is also a compound that produces agglomerations of particles, especially in their dry state, but also in dispersion in water. Thus, three compounds were identified that induce coagulation and flocculation of particles in aqueous dispersion, as well as agglomeration/aggregation in a solid state (dust binder). The agglomerations are also identified on the SEM images (Figure 5) of the solid materials resulting after the drying of the foam and the liquid (water with impurities).

Binding compounds in the form of aluminates are therefore identified by the 3854 cm^−1^ [11] liquid and foam) and 3615 cm^−1^ (foam) [11] hydroxyl-specific vibrations when bonding with aluminum in aluminates (Al–OH–Al) and the 614 cm^−1^ (liquid) band of Al-O deformation vibrations [8,19]. Sulfate ions denoted by the bands of 1627 cm^−1^ (liquid) and 1631 cm^−1^ (foam) are assigned to aluminum sulfate and ferrous sulfate with vibration modes within this range [18]. Surfactants from detergent-type cleaning products are noticed through the bands at 1424 cm^−1^ (liquid) and 1430 cm^−1^ (foam) assigned to –SO_2_ stretching asymmetric vibrations; (S–)CH_2_ deformation (usually at 1425 cm^−1^), (S–)CH_3_ deformation asymmetric, as well as the band at 1376 cm^−1^ (liquid) specific to R–SO_2_–OR structures; the bands at 877 cm^−1^, 798–775 cm^−1^, 712 cm^−1^ of S–O stretching vibration modes in sulfoxides, sulfones, sulfates and sulfites [17]; and 698 cm^−1^ (liquid and foam) of S–S stretching in sulfides and 557 cm^−1^ of C–S stretching. In the same range of 1424 cm^−1^ (liquid) and 1430 cm^−1^ (foam), carbonate groups may be assigned [18]. Under the effect of detergents to reduce the surface tension and allow the dispersed molecules to get close, the binding/flocculation/coagulation agents such as aluminates, aluminum and calcium sulfates, as well as calcium chloride, enter into Van der Waals interactions, hydrogen bridges and bonds coordination with organic molecules, but also with dust particles. It is observed in this sense by correlating the spectral lines in Figure 3 with the elemental composition in Figure 4 and Table 3, the presence of amino acids denoted by 3431 cm^−1^ and 3413 cm^−1^ bands of OH in COOH group and NH stretching vibrations; 2956 cm^−1^, 2920 cm^−1^, 2852 cm^−1^ (liquid) and 2960 cm^−1^, 2924 cm^−1^, 2852 cm^−1^ bands of –C–H bonds in aliphatic compounds, as well as 1874 cm^−1^, 1796 cm^−1^ bands assigned to NH and C=O groups in amides and carboxylic acids [17,20]. Silicates, silicon dioxide SiO_2_ and also silanol groups Si-OH are denoted in liquid dried sample by the bands at 3746 cm^−1^ (Si–OH), 2352 cm^−1^ (Si–H) 1093 cm^−1^ (Si–Si), 1029 cm^−1^ (Si–OH) and in foam dried sample by the bands at 2352 cm^−1^ (Si–H), 1084 cm^−1^ (Si–Si) and 1036 cm^−1^ (Si–OH) [16,17].

These compounds are “assembled” by the binding agents and structured in agglomerates. Sodium and calcium Ferro cyanides are also observed by the 1989 cm^−1^; 1966 cm^−1^; and 1936 cm^−1^ (liquid) bands correlated with the bands from 1627 cm^−1^ (liquid) and 1631 cm^−1^ (foam). The presence of cyanides indicates pollution by burning fossil fuels when HCN results, in this case subject to transformations to Ferro cyanides due to environmental conditions induced by the use of coagulants. This means that the water aerosols at one time had a content of both iron ions and hydrogen cyanide, which allowed the formation of the cyanide, as recent studies demonstrated that metal ions can react with the hydrogen cyanide adsorbed onto liquid water aerosols, leading to metal-cyanide complexes [21].

The analysis presented on both foam and liquid separately is to observe the distribution of the component phases in the aggregate structures under the influence of binding agents (aluminates, aluminum sulfate, calcium chloride). It is thus observed that there is a somewhat identical distribution of compounds between the two phases, foam and liquid, even if they are somewhat different in intensity/concentration. In fact, the non-homogeneous character in the distribution of the compounds is also found inside the same phase, as it results from the EDX analysis, depending on how the coagulation took place and which substances were identified in one area or another (Figure 4).

Coagulated structures with sizes of 100 μm or more are observed on the SEM images of Figure 4a,c, and similar “aerated” morphology both for the material obtained from the liquid dried sample and from the foam dried sample (Figure 5). This indicates the friability of the coagulated structures and their tendency to segregate into small particles under friction. Based on the EDX analysis, the binder based on aluminum that was used is more likely to be the aluminum sulfate, and less of aluminate (if any) because sodium is missing from the elemental composition. The FTIR spectra (Figure 3) and Table 2 indicate that calcium carbonate and aluminum sulfate can combine in certain proportions, leading to the formation of aluminum hydroxide with release of carbon dioxide inducing the foaming effect. Since at the time of sample collecting, the reaction was in progress (a fact denoted by abundant foam), the formed aluminum hydroxide was also identified in the FTIR spectra, as can be seen from Table 2. 

The investigation herein shows how, through the washing process, compounds in the form of dust and/or other waste were entrained on the city streets. The washing operation was not followed by the suction of the washing waters; they spread along the streets, on the green spaces and on the buildings, with part spilling into the collecting street canals with possible infiltration in the water pipes for the domestic supply water [8], and also by evaporation and dust dispersion in the air, enhancing the range of chemical species polluting the atmosphere [22,23,24,25]. 

The most affected areas by this kind of pollution event are those with high traffic. Taking this aspect into consideration, it is not surprising to find the “fingerprint” of the dust binding compounds in the dust samples collected from buildings. Regarding this aspect, the FTIR spectra of the samples collected from the surface of the precinct wall of Golia (the Golia Wall samples denoted as Wst-b, Wd-d, Wm, and S) Figure 3b–e show contamination with the chemical compounds identified in the samples collected from the streets after the cleaning process (denoted as “liquid” and “foam”), as per Figure 3a. Aluminates and aluminum sulfate specific bands are evidenced in the material of the Golia Wall samples, as well as organic compounds such as amines, amides, carboxylic acids and others (Table 2) bound in coagulated structures. 

Among all the chemical species, aluminates and aluminum sulfate, as well as calcium chloride, are of the most interest to our study. These are the markers that allow tracking the environmental side effects induced by the dust binding procedure applied on municipal streets. The “spreading” of the particles resulting from the dust binding process is facilitated by the intense traffic on the streets and boulevard surrounding the precinct wall of the Golia Monastery. This explains the high similarity between the “Golia Wall samples” and the “Streets samples”, mainly between the Wst-b and the foam spectra, which exhibit identical vibration bands in the fingerprint zone.

Following the tracking marker element Aluminum in the Golia Wall samples as the EDX analysis shows (Table 4), there is the indication of contamination induced by the particles formed on the street as a result of the dust binding process, followed by the crushing of the aggregates through the mechanical action induced by the intense traffic and favored by the wind conditions and the circulation of air currents in the area. 

Morphological analysis in the SEM images (Figure 5), together with maps of element distribution on the analyzed areas, provide structural images of particles of 10 μm or less and micro-aggregates of over 100 μm with content of aluminum. As coagulant agent and the element constituents of chemical compounds are involved in physical bonds (Van der Waals interactions and H-bonds) with aluminum, there are therefore indications that the particles related to the coagulants used to clean the streets and carried by the wind ended up being partially deposited on the Precinct Wall of the Golia Monastery.

### 3.2. Coloristic Analysis of Golia Wall Sampling Spots

**Figure 6:**
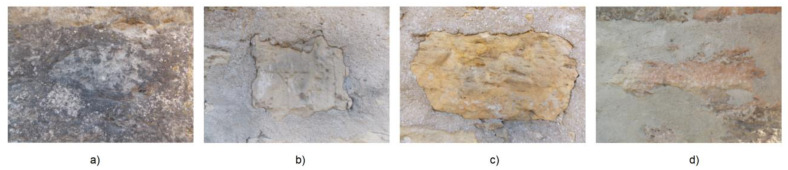
The sampling areas on the precinct Wall of Golia Monastery of Iasi: Wst-b (**a**); Wd-d (**b**); Wm (**c**); and S (**d**).

The coloristic test conducted in CIELAB coordinates (L*a*b*) on the Golia Wall sampling and neighboring spots aims to quantify color and its lightness deviation level on the analyzed walls (Figure 6a–d). The parameters used are $\Delta a^{*}=\left(a_{\mathrm{CLSS}}^{*}-a_{m}^{*}\right)$ for red-green deviation (a* > 0 for red; a* < 0 for green), $\Delta b^{*}=\left(b_{\mathrm{CLSS}}^{*}-b_{m}^{*}\right)$ for yellow-blue deviation (b* > 0 for yellow and b* < 0 for blue) and $\Delta E=\sqrt{{\left(\Delta L^{*}\right)}^{2}+{\left(\Delta a^{*}\right)}^{2}+{\left(\Delta b^{*}\right)}^{2}}$ for total color deviation (Table 5). 

Nonhomogeneous color is obvious, at the same the level of deterioration. The coloristic evaluation in this case is a factor to quantify the damages as an impact of external parameters of humidity and temperature, and of the particles deposited on the wall from mainly anthropogenic sources. The reference for coloristic deviations was chosen as the sampling spot Wd-d as being dry at the moment when the samples were collected.

**Figure 7:**
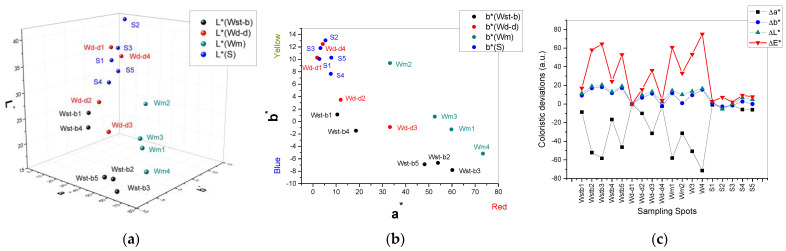
Coloristic measurements on the sampling spots and their neighboring spots: 3D diagram of L*a*b*coloristic components of the measured points (**a**); 2D diagram of ab* coloristic components of the measured points (**b**); and diagram of coloristic deviations Δa*, Δb*, ΔE* (**c**).

The diagrams (a) and (b) of Figure 7 indicate the coloristic components of each sampling area as it follows: gray and purple dark colors for Wst-b; gray and yellow light color (giving the aspect of a yellowish color) for Wd-d; yellow slightly to orange due to the red component for Wm and also gray and purple spots on the cement surrounding Wm sampling area; and brownish yellow in the S sampling area. Analyzing the coloristic deviations Δa*, Δb*, ΔE* of the measured points on the Golia Wall sampling spots, the lowest levels are noticed for the southern area (S sampling area), while the western area is the most affected by such deviations. 

The highest influence is given by the component Δa*. Because the green component was not detected on either one of the sampling areas Figure 7a,b, the high influence in Δa* is related to the red component. Given the iron element detected in EDX analysis of all Golia Wall samples (Table 4), as well as in the street samples, different iron oxides and iron hydroxide can be assigned to each sampling spot. The most interesting is the purple color of the most exposed of all Golia Wall sampling spots analyzed herein. This color could be assigned to Fe_2_O_3_ of coarser particles that were deposited on the wall with the dust from the streets. 

The size of the particles of iron oxide in this case could be explained by the use of dust binders. The high content in carbon (43.87% mass or 54.04% atomic) also indicates a contribution to the darkness of the color by the soot resulting from the intense traffic. Since there was no water infiltration found in the walls of the building, the color changes cannot be attributed to efflorescence or subflorescence, and pollution with particles from the street and the atmosphere remains the only cause to be considered.

The coloristic analysis therefore provided supplementary information on this study regarding pollution effects. It is also an important tool in the restoration procedures, including regaining the initial color of the edifice, but also in finding parameters and solutions for the removal of the coating impurities. This responds to a previous study on the Precinct Wall of Golia Monastery reported by Pelin in 2019, Germany [26], which states that the respective construction has been in a state of preservation apparently satisfactory from a functional point of view, but precarious in terms of the quality and condition of the constitutive lithic material with significant degradation and damages, which generate irreversible material losses with passing years without concrete restoration measures, or at least preservation.

### 3.3. Meteorological and Atmospheric Quality Context

Meteorological parameters such as humidity and wind contributed to the morphological changes of the particulate matter and aggregates, resulting in the dust binding process and the spread conditions of those. On 10 March 2021, the data regarding the wind rose show an east-southeast predominant wind direction correlated with a predominantly high speed exceeding 6 m/s and a mean speed of 5.2 m/s. In terms of wind intensity, the data provide characteristics of the day as being active with frequency stability classes’ distribution within an unstable and very unstable range. Following the analysis of the data on the site [9], on 14 April 2021 the wind blew to the west in almost 30% of the day with a predominant speed of between 1–2 m/s, with a predominant stability class of 3, falling predominantly unstable and very unstable. The mean wind speed was 2.8 m/s.

In the atmosphere, between 9 and 10 March 2021, the high wind speed influenced a trend of increased AOD (aerosol optical depth) level 500 nm from 0.15 to 0.27, correlated with a slight increase of the AE (Angstrom Exponent) 440–870 nm level from 1.4983 to 1.6317, characterizing fine particles.

Weak correlation was observed between temperature, relative humidity and AOD, while a strong correlation was observed between wind speed and AOD. The dry soil maintained due to low rainfall and high levels of wind speed favored the loading of the atmosphere with the coarse dust aerosols. 

Moreover, on 14 April 2021, there was an increase in the 500 nm AOD level correlated with a decrease in the AE 440–870 nm level [10], with this relationship between the two parameters indicating a high abundance of coarse particles detected in the atmosphere.

### 3.4. Effects of Streets Washing with Aqueous Solutions Containing Dust Binders

Tracking dust binding effects induced by the cleaning procedure, the marker consisting of aluminum showed that similar chemical compounds and structures ended up on the Golia Wall.

**Figure 8:**
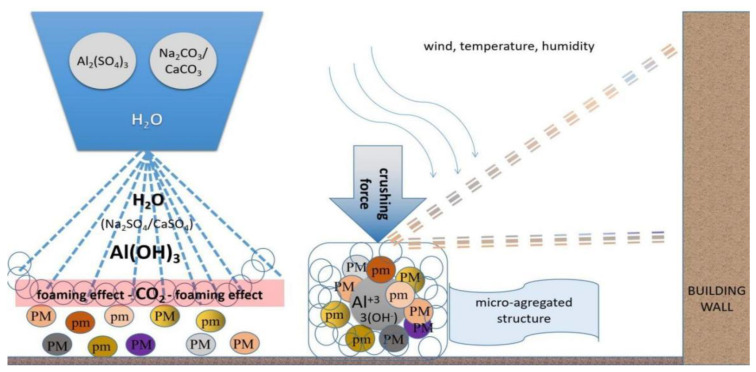
Schematic representation of the physico-chemical processes of dust binding process on the streets and deposition on the building’s walls.

Summarized physico-chemical processes are schematically represented in Figure 8. In the cleaning solution, aluminum hydroxide (Al(OH)_3_) results from aluminum sulfate (Al_2_(SO_4_)_3_ and carbonates (Na_2_CO_3_/CaCO_3_), with a foaming effect due to carbon dioxide (CO_2_) being released during the reaction. The dust binding process due to aluminum hydroxide leads to aggregated structures of particulate matter of different sizes (noted as PM and pm). Under mechanical processes and forces due to high traffic, the micro-aggregated structures formed by dust binding are crushed into smaller micro-aggregates. Finally, the meteorological conditions and the small distance from the street to the wall may favor deposition of the crushed particles of micro-aggregates.

## 4. Conclusions

The effects of using dust binders in different activities, including but not limited to the dust cleaning procedures on urban streets, can be reflected by the deposition of chemically and structurally similar particulate matter on the walls of the buildings. 

The issues arising from the deficient application of some procedures that are still the subject of study can bring more damage than benefits. On the other hand, the need to establish measures to restore old buildings is indicated, and how certain analysis techniques can be used in this regard. 

Cleaning techniques and technologies need to be improved in order to avoid adding more pollutants to the air, and with deposits on buildings or penetrating inside them, infiltrating the drinking water network will lead to a worsening of the situation from the perspective of pollution in the urban environment. Removing procedures like vacuum cleaning are required to be used with the aim to collect the resultant dust binding material. Another alternative way to reduce the environmental impact is to limit the speed of vehicles to no more than 25 Km/h, and/or to ban traffic of heavy vehicles. Another possible intervention is to ban traffic of all vehicles in the vicinity of the Monastery.

## Figures and Tables

**Table 1:** Distances from the Wall Sampling spots to the Street.

Golia Wall Sampling Spot	Distance between the Sampling Area and the Street
Wst-b	190 cm
Wd-d	305 cm
Wm	308 cm
S	1315 cm

**Table 2:** Functional groups of the FTIR spectra of Figure 3. * there is no vibration in that range.

Spectral Vibration Bands (cm^−1^)						Functional Groups	References
Street Samples		Golia Wall Samples					
Liquid	Foam	Wd-d	Wm	Wst-b	S		
3854	3854	3854	3854	3854	3854	Al–OH–Al stretching modes	[11]
3746	*	3746	3746	3746	3746	Si–OH stretching modes	[12,13,14,15,16]
*	3615	3615	3627	*	3627	Al–OH–Al stretching modes	[11]
*	*	*	3569	*	3569	OH stretching in alcohols free	
3431–3413	3441–3413	3428–3401	3428–3392	3428–3388	3428	OH stretching vibrations N–H stretching vibrations in primary amides	[8,17]
*	*	3274–3247–3211	*	*	3392	N–H stretching	[17]
2956	2960	2976	2980	2960	2980	C–H aliphatic stretching; SH thiol group stretching in aliphatics	[17]
2920	2924	2944	2944	2935	2944	C–H aliphatic stretching	[17]
2852	2852	2877–2822	2877	2881	2877	C–H aliphatic stretching	[17]
*	*	*	2764	*	2764	C–H aliphatic stretching in aldehydes	[17]
*	*	2591	2578	*	2578	S–H thiol	[17]
2510	2504	2514	2510	2510	2510	P–H stretching O–H in COOH	[17]
*	*	*	2428	*	*		
2352	2352	2383	2379	*	2379–2351	O=C=O stretching (carbon dioxide) Si–H stretching	[17]
*	1989; 1966; 1936	*	*	*	*	FerrocyanidesSodium ferrocyanide Na_4_Fe(CN)_6_ Calcium ferrocyanide Ca_2_Fe(CN)_6_	[18]
1874; 1796	*	1796	1765	*	1795	Amino NH and carbonyl C=O groups in amides and aminoacids	[17]
1650; 1627	1631	1636	1632	1650	1632	Amino NH and carbonyl C=O groups in amides and aminoacids; Ferrous sulfate FeSO_4_; Aluminum sulfate Al_2_SO_4_ Calcium sulfate CaSO_4_∙2H_2_O Ferrocyanides	[17,18]
1424	1430	1428	1442	1430	1428	Calcium carbonate CaCO_3_ –SO_2_ stretching asymmetric vibrations (S–)CH_2_ deformation (1425 cm^−1^); (S–)CH_3_ deformation asymmetric	[17,18]
1376	*		1379	*	*	R–SO_2_–OR	[17]
*	*	1170	1170	*	1170	C–O stretching in alcohols	[17]
1093	1084	1084	1084	1084	1084	C–O stretching Si–O–Si SO_4_^2−^ in Ferrous sulfate SO_4_^2−^ in Aluminum sulfate	[16,17,18]
1029	1036	1034	1038	1038	1038	Si–OH deformation modes	[16,17]
877	872	872	876	872	876	S–O stretching in sulfoxides, sulfones, sulfates and sulfites	[17]
798–775	798–775	790–771	795–776	798–775	795–784	S–O stretching in sulfoxides, sulfones, sulfates and sulfites	[17]
712	712	713	718	712	718	S–O stretching in sulfoxides, sulfones, sulfates and sulfites	[17]
698	698	668	668	698	668	S–S in sulfides	[17]
*	*	631	627	*	627		
614	*	*	*	*	*	Al–O stretching vibrations	[8,19]
557	557	*	*	*	*	C–S stretching	[17]

**Table 3:** Street samples elemental composition in EDX analysis. The sign “*” means here “missing element” in the elemental composition of the sample, i.e., the samples do not contain Potassium in this case and the samples Foam 1 does not contain Copper.

Element	Mass Norm. [%]				Atom [%]			
	Liquid 1	Liquid 2	Foam 1	Foam 2	Liquid 1	Liquid 2	Foam 1	Foam 2
Oxygen	36.29	33.41	31.59	28.05	47.91	46.25	41.81	36.93
Carbon	7.7	6.46	11.36	14.41	13.54	11.91	20.02	25.27
Nitrogen	0.81	0.78	0.2	0.9	1.22	1.23	0.3	1.35
Calcium	5	6.91	9.35	6.57	2.63	3.81	4.94	3.46
Silicon	30.49	27.28	27.87	18.61	22.93	21.5	21.01	13.96
Iron	4.11	6.26	6.89	6.24	1.55	2.48	2.61	2.35
Phosphorus	0.08	0.29	0.34	0.36	0.06	0.21	0.23	0.24
Potassium	*	*	*	*	*	*	*	*
Aluminum	4.64	5.17	8.2	8.53	3.64	4.24	6.44	6.66
Copper	0.25	0.51	*	0.58	0.08	0.18	*	0.19
Chlorine	8.93	11.09	2.16	12.05	5.32	6.92	1.29	7.16
Sulfur	1.7	1.84	2.04	3.7	1.12	1.27	1.35	2.43
	100	100	100	100	100	100	100	100

**Table 4:** Golia Wall samples. The sign “*” is for elements that are missing in some of the samples.

Element	Mass Norm.				Atom			
	[%]				[%]			
	West Down Dry (Wd-d)	West Middle (Wm)	West—Support Pole towards the Street—Black (Wst-b)	South (S)	West Down Dry (Wd-d)	West Middle (Wm)	West—Support Pole towards the Street—Black (Wst-b)	South (S)
Oxygen	50.65	56.09	43.09	53.23	55.49	65.14	39.83	61.94
Carbon	21.56	11.95	43.87	13.92	31.47	18.48	54.04	21.58
Calcium	21.04	21.06	3.34	25.15	9.2	9.76	1.23	11.68
Silicon	4.67	7.73	7.73	5.6	2.92	5.11	4.07	3.71
**Iron**	**1.28**	**1.18**	**0.9**	**1.02**	**0.4**	**0.39**	**0.24**	**0.34**
Potassium	*	0.74	*	*	*	0.35	*	*
**Aluminium**	**0.8**	**0.69**	**1.07**	**1.08**	**0.52**	**0.48**	**0.59**	**0.75**
Chlorine	*	0.56	*	*	*	0.29	*	*
	100	100	100	100	100	100	100	100

**Table 5:** CIELAB coordinates (L*a*b*) on the Golia Wall sampling and neighboring spots. Bold represents sampling points, while others are adjacent points.

		L*	a*	b*	ΔL*	Δa*	Δb*	ΔE*
Wst-b	**Wstb1**	**24.35**	**10.51**	**1.13**	**11.38**	**−8.71**	**9.15**	**17.00**
	Wstb2	16.76	53.93	−6.66	18.97	−52.13	16.94	58.00
	Wstb3	15.35	60.13	−7.80	20.38	−58.33	18.08	64.38
	Wstb4	22.56	18.55	−1.48	13.17	−16.75	11.76	24.34
	Wstb5	16.70	48.17	−6.88	19.03	−46.37	17.16	52.98
Wd-d	**Wd-d1**	**35.73**	**1.80**	**10.28**	**0.00**	**0.00**	**0.00**	**0**
	Wd-d2	26.10	11.97	3.50	9.63	−10.17	6.78	15.56
	Wd-d3	22.56	33.24	−0.89	13.17	−31.44	11.17	35.87
	Wd-d4	33.57	4.26	12.47	2.16	−2.46	−2.19	3.94
Wm	**Wm1**	**21.43**	**59.76**	**−1.26**	**14.30**	**−57.96**	**11.54**	**60.80**
	Wm2	25.59	33.25	9.40	10.14	−31.45	0.88	33.06
	W3	22.14	52.58	0.79	13.59	−50.78	9.49	53.42
	W4	19.29	73.36	−5.16	16.44	−71.56	15.44	75.03
South	**S1**	**33.05**	**2.77**	**10.07**	**2.68**	**−0.97**	**0.21**	**2.86**
	S2	41.33	5.38	13.06	−5.60	−3.58	−2.78	7.20
	S3	35.40	3.23	11.81	0.33	−1.43	−1.53	2.12
	S4	29.05	7.70	7.67	6.68	−5.90	2.61	9.29
	S5	30.93	7.91	10.27	4.80	−6.11	0.01	7.77

## Data Availability

Not applicable.
